# Should Splenic Hilar Lymph Nodes be Dissected for Siewert Type II and III Esophagogastric Junction Carcinoma Based on Tumor Diameter? A Retrospective Database Analysis

**DOI:** 10.1097/MD.0000000000003473

**Published:** 2016-05-27

**Authors:** Chen-Bin Lv, Chang-Ming Huang, Chao-Hui Zheng, Ping Li, Jian-Wei Xie, Jia-Bin Wang, Jian-Xian Lin, Jun Lu, Qi-Yue Chen, Long-Long Cao, Mi Lin, Ru-Hong Tu

**Affiliations:** From the Department of Gastric Surgery, Fujian Medical University Union Hospital, Fuzhou, Fujian Province, China.

## Abstract

The aim of the study is to identify the value of a spleen-preserving No. 10 lymphadenectomy (SPL) for Siewert type II/III adenocarcinoma of the esophagogastric junction (AEG).

From January 2007 to June 2014, 694 patients undergoing radical total gastrectomy for Siewert type II/III AEG were analyzed. Oncologic outcomes were compared between SPL and no SPL (No. 10D+ and No. 10D–) groups.

The incidence of No. 10 lymph node metastasis (LNM) was 12.3%. No significant differences in the incidence of No. 10 LNM were found between Siewert type II AEG with tumor diameters of <4 cm and ≥4 cm (*P* = 0.071). However, Siewert type III AEG with a tumor diameter ≥4 cm showed a significantly higher frequency of No. 10 LNM compared with a tumor diameter <4 cm (*P* < 0.001). The No. 10D+ group had superior 3-year overall survival (OS) and disease-free survival (DFS) rates compared with the No. 10D− group (*P* = 0.030 and *P* = 0.005, respectively). For patients with Siewert type II and type III AEG with a tumor diameter <4 cm, the 3-year OS and DFS rates were similar between the 2 groups. However, the No. 10D+ group had better 3-year OS (66.6% vs 51.1%, *P* = 0.019) and DFS (63.2% vs 45.9%, *P* = 0.007) rates for Siewert type III AEG with a tumor diameter ≥4 cm. A multivariate Cox regression showed that SPL was an independent prognostic factor in Siewert type III AEG with a tumor diameter ≥4 cm.

SPL may improve the prognosis of Siewert type III AEG with a tumor diameter ≥4 cm, whereas SPL may be omitted without decreasing survival in patients with Siewert type II or type III AEG with a tumor diameter <4 cm.

## INTRODUCTION

The incidence of adenocarcinoma of the esophagogastric junction (AEG) is increasing.^1,2^ Siewert and Stein^3^ classified AEG into 3 subgroups according to the location of the tumor's epicenter. Perigastric regional nodal metastasis comprises the majority of Siewert type II/III AEG cases, and the incidence of No. 10 lymph node metastasis (LNM) is reported to be 4.8% to 15.0%.^4–7^ Biological behavior of AEG differs among types and particularly with regard to the incidence of No. 10 LNM.^8,9^ Therefore, the value of a No. 10 lymphadenectomy for Siewert type II and III AEG is fairly controversial.^9,10^ Several recent studies have indicated that tumor diameter not only is prognostic in gastric cancer but also correlates with LNM.^11,12^ The Japanese Gastric Cancer Association and the Japan Esophageal Society assessed the status of LNM in AEG with a tumor diameter <4 cm and developed a flow diagram to identify the extent of the lymphadenectomy. They did not recommend a No. 10 lymphadenectomy in the 4th edition of Japanese gastric cancer treatment guidelines.^13^ However, whether a No. 10 lymphadenectomy should be performed for AEG with a tumor diameter ≥4 cm was not addressed. Therefore, this study sought to investigate the survival benefits of a spleen-preserving No. 10 lymphadenectomy (SPL) for Siewert type II and III AEG with tumor diameters of <4 cm and ≥4 cm.

## MATERIAL AND METHODS

### Patients

From January 2007 to June 2014, a prospectively maintained database identified 694 patients with Siewert type II/III AEG who underwent a transabdominal radical total gastrectomy. Based on whether they underwent a spleen-preserving No. 10 lymphadenectomy (SPL), patients were divided into No. 10D+ (n = 293) and No. 10D– (n = 401) groups. The necessity of a No. 10 lymphadenectomy was evaluated according to the preoperative examination and intraoperative exploration by the same group of surgeons, all of whom had significant experience in laparoscopic radical gastrectomy. A No. 10 lymphadenectomy was also performed by the same group of surgeons. The inclusion criteria were as follows: histological confirmation of Siewert type II or III AEG; pathological confirmation of stage T1 to T4a; no evidence of distant metastasis; a completely transabdominal approach; and curative R0. The exclusion criteria included preoperative radiotherapy or chemotherapy, combined major organ resection, presence of a tumor invading >3 cm into the esophagus, or incomplete pathological data. Preoperative imaging studies, including computed tomography (CT) scanning, ultrasonography (US) of the abdomen and endoscopic US, were routinely performed following an endoscopic examination. The severity of complications was classified according to the Clavien–Dindo grade, and major complications were at least grade III.^14^ Staging was determined according to the 7th edition of the International Union Against Cancer (UICC) TNM classification.^15^ Adjuvant chemotherapy with 5-fluorouracil (5-FU)-based regimens (generally 5-FU with cisplatin) was recommended for every patient with positive nodal disease or advanced cancer. Written informed consent was obtained from all patients prior to surgery. This study was approved by the institutional review board of Fujian Medical University Union Hospital.

### Surgical Procedures

All patients were treated with a radical total gastrectomy. In terms of the lymphadenectomy, a D2 lymphadenectomy including SPL was performed on patients in the No. 10D+ group.^16–18^ In the No. 10D– group, the extent of the lymphadenectomy was the same as in the No. 10D+ group but without the SPL. Specifically, after entering the retropancreatic space from the superior border of the pancreas, the left gastroepiploic vessels were sufficiently exposed at the tail of the pancreas and the lower pole of the spleen. These vessels were sufficiently vascularized and dissected. However, the remaining short gastric vessels were divided at their roots directly to the left cardiac region. The splenic vessels were not vascularized, and the lymph nodes in this region were not dissected. An intraoperative frozen section analysis was performed during every operation, and the adequacy of the extent of esophageal resection was confirmed.

### Measurement of Tumor Diameter

The tumor diameter was measured according to the Japanese Classification of Gastric Carcinoma.^19^ Briefly, the resected stomach was opened along the greater or lesser curvature to expose the entire mucosa. Then the opened stomach was examined macroscopically with the mucosal side up, and the diameter and thickness of each tumor were recorded. The longest diameter was used in the present study.

### Follow-Up

Patients were followed up every 3 months for 2 years and then every 6 months from 3 to 5 years. Most routine follow-up consisted of laboratory tests, chest radiography, abdominopelvic ultrasonography, or computed tomography. Endoscopic examination was performed every 12 months. Overall survival (OS) was calculated from the day of surgery until death or until the final follow-up date of June 2015, whichever occurred first. Recurrence was diagnosed based on radiological or histological signs of disease. Disease-free survival (DFS) was calculated from the day of surgery to the day of recurrence or death.

### Statistical Analysis

The chi-square test or Fisher's exact test was used to compare categorical variables, and Student's *t* tests were used to evaluate continuous variables. Multivariate analysis that used binary logistic regression models was performed to further evaluate factors found to be significantly prognostic on univariate analysis. Cumulative survival rates were compared using the Kaplan–Meier method and the log-rank test. All statistical analyses were performed using SPSS v. 18.0 for Windows (SPSS Inc., Chicago, IL). Values of *P* < 0.05 were considered to be statistically significant.

## RESULTS

### Patient Characteristics

The general clinicopathologic characteristics are summarized in Table 1. There were 293 patients in the No. 10D+ group and 401 in the No. 10D– group. Of the 694 patients, 304 (43.8%) had Siewert type II AEG and 390 (56.2%) had type III AEG. The mean tumor diameter was 4.9 ± 2.2 cm, and 71.9% (499/694) of patients had a tumor diameter ≥4 cm. In patients with a tumor diameter <4 cm, the body mass index (BMI), Siewert classification, approach and N stage differed significantly between the No. 10D+ and No. 10D– groups (*P* < 0.05). In patients with a tumor diameter ≥4 cm, the No. 10D+ group had a significantly advanced T stage compared to the No. 10D– group (*P* = 0.048). Postoperative complications occurred in 94 (13.5%) patients, and 30 patients (4.3%) experienced a major postoperative complication. The No. 10D+ group had significantly more major complications (8.6% vs 3.0%, *P* = 0.006) for patients with tumor diameter ≥4 cm (Table 1).

**TABLE 1:**
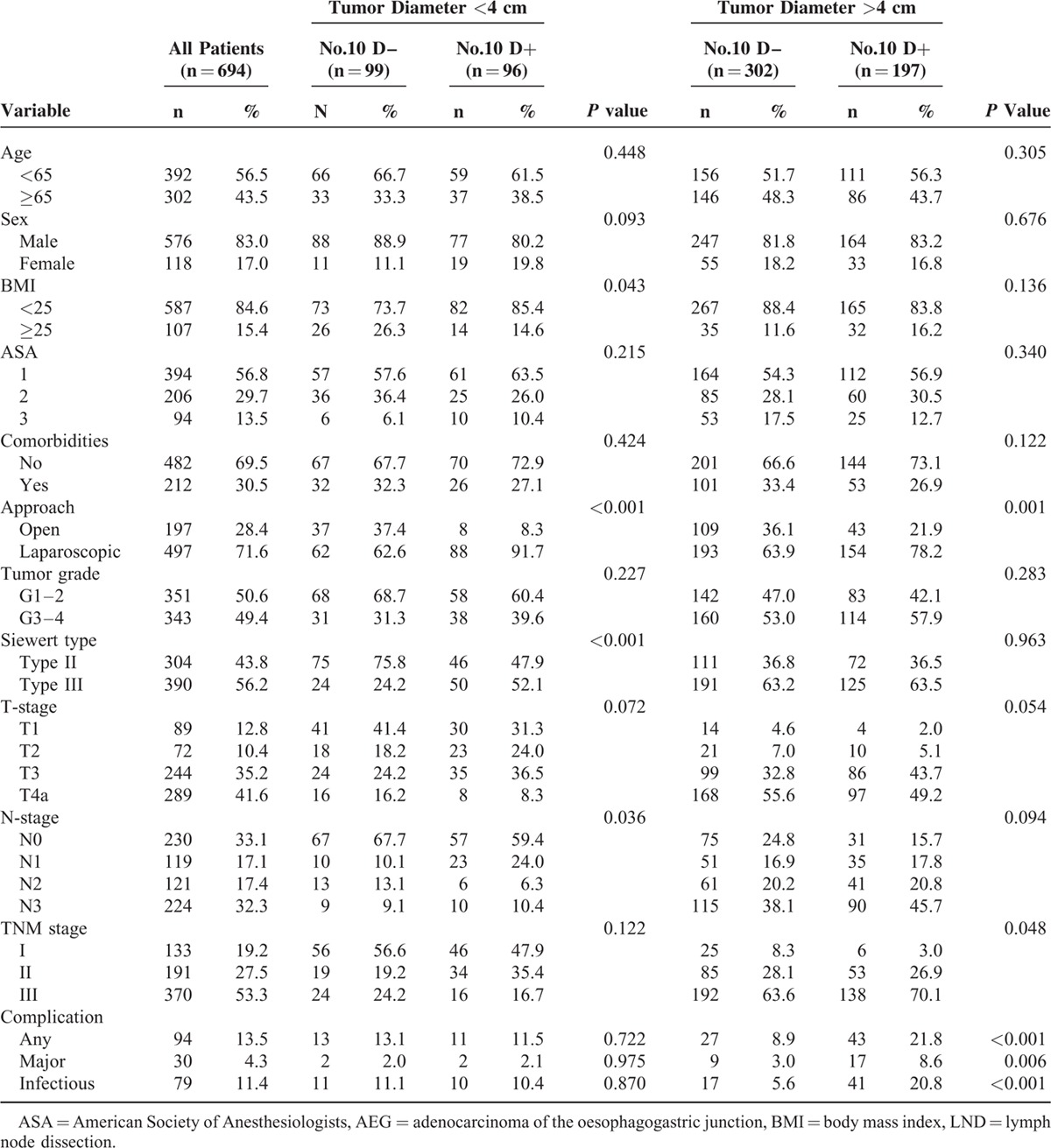
Clinicopathologic Features of All Patients With Siewert Type II and III AEG, Stratified by Tumor Diameter and No.10 LND Status

### Incidence of No. 10 LNM in the No. 10D+ Group

**FIGURE 1:**
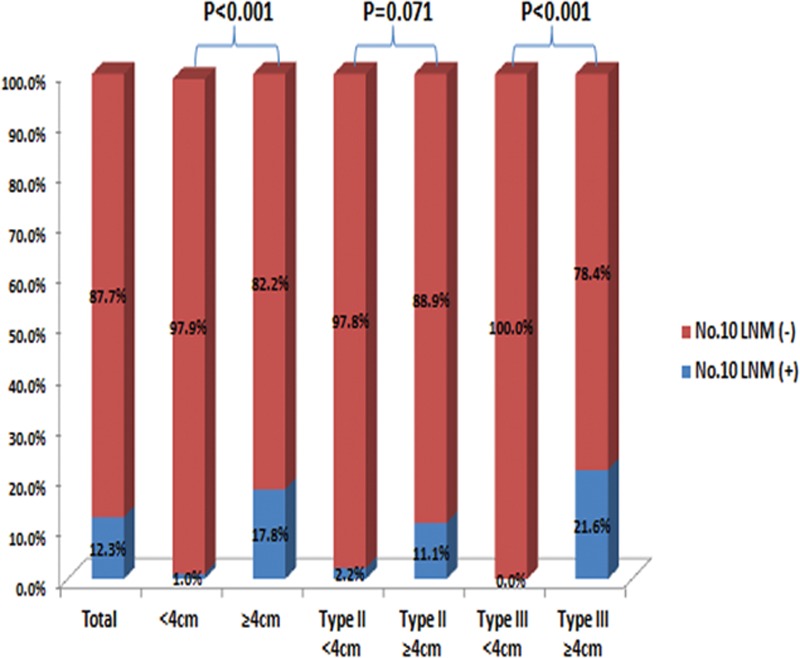
Incidence of No. 10 LNM in the No. 10D+ group.

Thirty-six (12.3%) patients exhibited No. 10 LNM. The incidences of No. 10 LNM were 7.6% in patients with Siewert type II AEG and 15.4% in patients with type III AEG (*P* = 0.046). A significantly higher rate of No. 10 LNM was observed in patients with a tumor diameter ≥4 cm than with those with a tumor diameter <4 cm (17.8% vs 1.0%, *P* < 0.001). A stratified analysis demonstrated that there were no significant differences in No. 10 LNM for Siewert type II AEG with tumor diameters of <4 cm and ≥4 cm (*P* = 0.071). However, Siewert type III AEG with a tumor diameter ≥4 cm showed a significantly higher rate of No. 10 LNM compared with a tumor diameter <4 cm (21.6% vs 0.0%, *P* < 0.001) (Figure 1). For patients with No. 10 LNM, the percentages of N1–3 were 11.1%, 11.1%, and 77.8%, respectively, and they were 21.0%, 17.1%, and 28.0% for patients without No. 10 LNM, respectively. N stage was significantly more advanced for patients with compared to without No. 10 LNM (*P* < 0.001).

### Oncologic Outcomes

**FIGURE 2:**
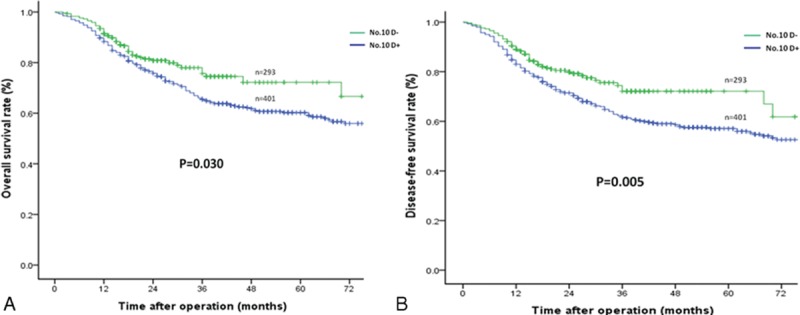
Comparison of the overall and disease-free survival rates between the No. 10D+ and No. 10D– groups: (A) overall survival outcomes and (B) disease-free survival outcomes.

**FIGURE 3:**
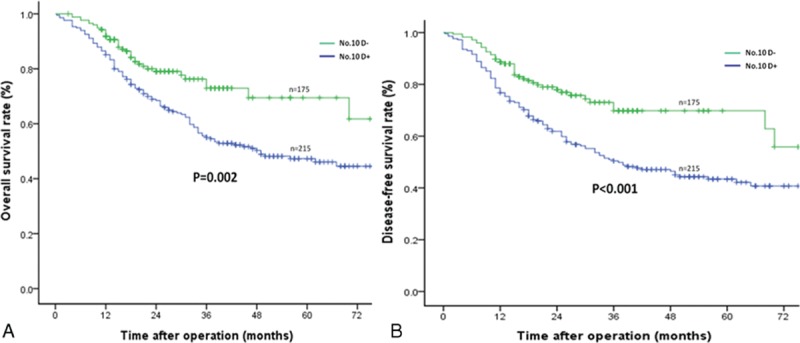
Comparison of the overall and disease-free survival rates of Siewert type III AEG between the No. 10D+ and No. 10D– groups: (A) overall survival outcomes and (B) disease-free survival outcomes. AEG = adenocarcinoma of the oesophagogastric junction.

The median follow-up period was 47 (range 11–100) months. The No. 10D+ group showed superior 3-year OS and DFS rates compared to the No. 10D– group (*P* = 0.030 and *P* = 0.005, respectively) (Table 2 and Figure 2A and B) . For patients with Siewert type II AEG, the 3-year OS and DFS rates were similar between the 2 groups (Table 2). However, the No. 10D+ group showed significantly longer 3-year OS (72.9% vs 55.1%, *P* = 0.002) and DFS (69.8% vs 50.4%, *P* < 0.001) in patients with Siewert type III AEG (Table 2, Figure 3A and B).

**TABLE 2:**
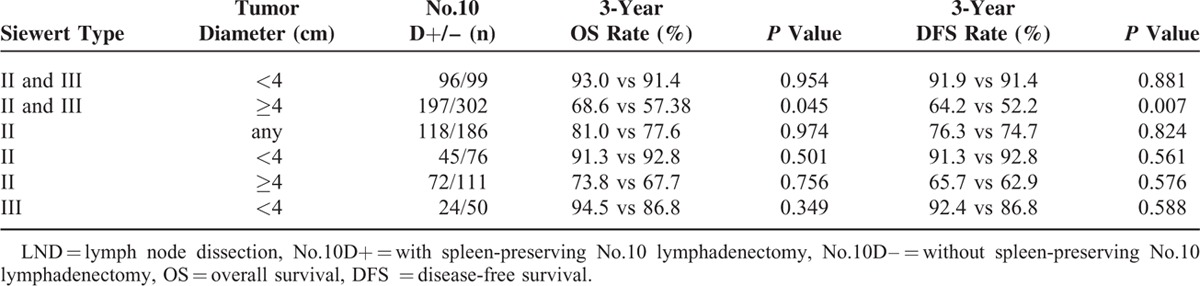
Interactions Between Tumor Diameter and Siewert Type on the Survival of Patients With/Without No.10 LND

### Stratified Analysis of Survival According to Tumor Diameter

**FIGURE 4:**
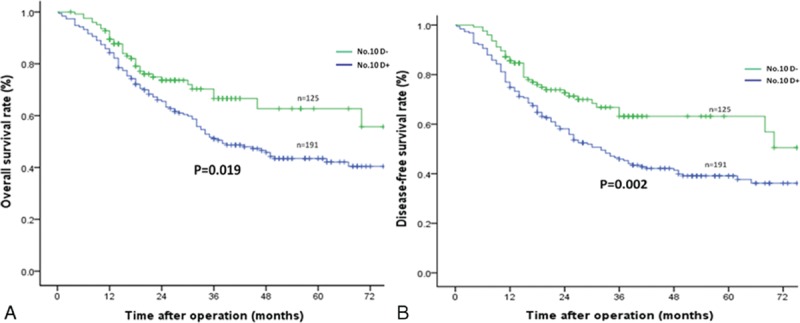
Comparison of the overall and disease-free survival rates of Siewert type III AEG with a tumor diameter ≥4 cm between the No. 10D+ and No. 10D– groups: (A) overall survival outcomes and (B) disease-free survival outcomes. AEG = adenocarcinoma of the oesophagogastric junction.

For Siewert type II AEG with different tumor diameters (<4 cm and ≥4 cm), the 3-year OS and DFS rates did not differ significantly between the No. 10D+ and No. 10D– groups (Table 2). In patients with Siewert type III AEG and a tumor diameter <4 cm, the differences in the 3-year OS and DFS rates between the 2 groups were not statistically significant (Table 2). However, the No. 10D+ group had better 3-year OS (66.6% vs 51.1%, *P* = 0.019) and DFS (63.2% vs 45.9%, *P* = 0.007) rates for patients with Siewert type III AEG and a tumor diameter ≥4 cm (Table 2 and Figure 4A and B).

### Univariate and Multivariate Survival Analyses and Clinicopathologic Characteristics of Patients With Siewert Type III AEG and a Tumor Diameter ≥4 cm

The clinicopathologic characteristics of patients with Siewert type III AEG and a tumor diameter ≥4 cm were comparable between the No. 10D+ and No. 10D– groups (Table 3). Factors potentially related to OS and DFS in the univariate analysis were No. 10 lymph node dissection (LND), age, tumor grade, T stage, and N stage (Table 3). The multivariate model identified No. 10 LND, age, T stage, and N stage as independent predictors of OS and DFS (Table 4).

**TABLE 3:**
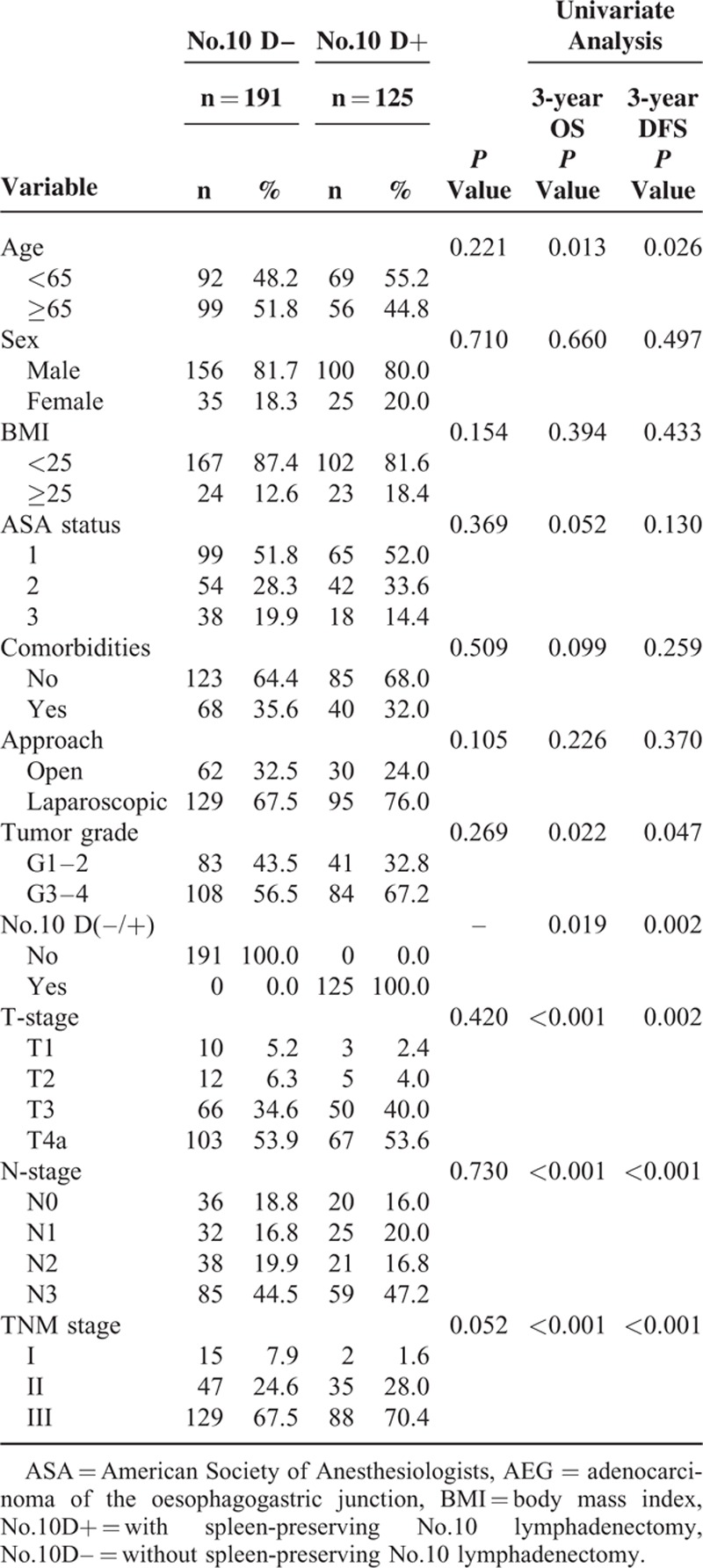
Clinicopathologic Features of Patients With Siewert Type III AEG With Tumor Diameter ≥4 cm, and Univariate Analysis of Risk Factors Associated With Survival

**TABLE 4:**
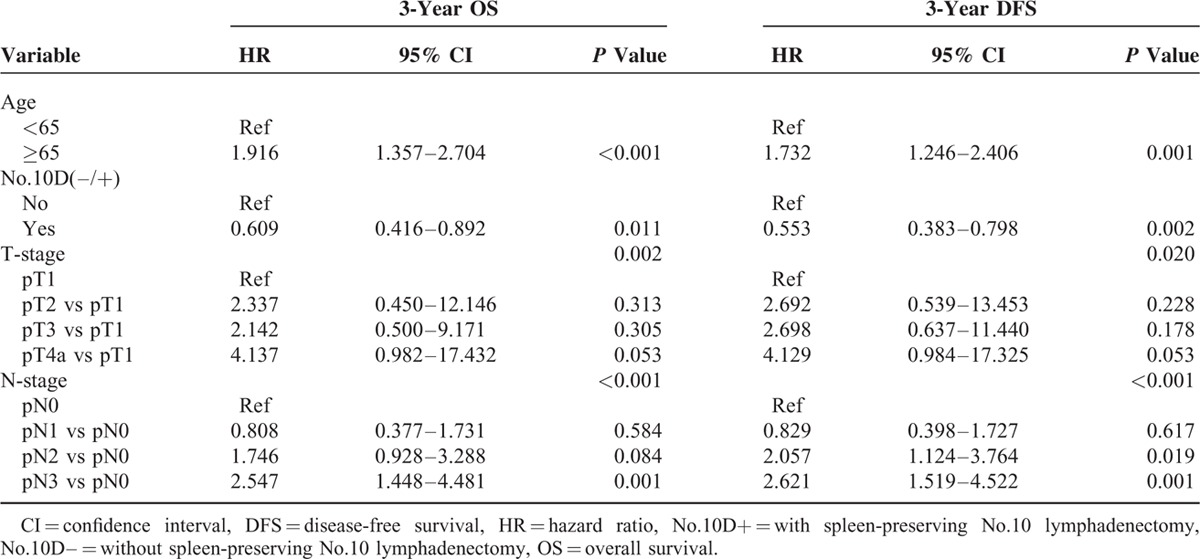
Multivariate Cox Regression Analysis of Risk Factors Associated With the Survival of Siewert Type III AEG with Tumor Diameter ≥4 cm

## DISCUSSION

Surgical management is the primary treatment of AEG. Perigastric regional nodal metastasis comprises the majority of AEG cases, and the transabdominal approach is recommended for Siewert type II/III tumors invading <3 cm into the esophagus according to the results of the JCOG 9502 trial.^20^ However, a small number of reports have focused on No. 10 LNM for type II and III AEG because of the particular anatomical location. Hasegawa et al^6^ retrospectively analyzed 121 patients with Siewert type II/III AEG and determined that the incidence of No. 10 LNM was 10.7%. The frequency of No. 10 LNM was 12.3% in our study. In addition, No. 10 LN metastatic rates differ among types. Hasegawa et al and Kakeji et al^6,21^ showed that the rate of No. 10 LNM in Siewert type II AEG was much lower than in type III AEG. In this study, the incidence of No. 10 LNM in Siewert type II AEG was 7.6%, which was also much lower than the rate of 15.4% in type III AEG (*P* = 0.046). It has been reported that tumor diameter in proximal gastric cancer correlates with No. 10 LNM.^22^ Shin et al^23^ indicated that the splenic hilar metastasis group in proximal gastric cancer had a higher proportion of tumor diameters >5 cm (82.9%). Fang et al^5^ found no incidence of No. 10 LNM in Siewert type II AEG with a tumor diameter <4 cm. The 4th edition of the Japanese gastric cancer treatment guidelines, published in 2014, also revealed a low incidence of No. 10 LNM in AEG with a tumor diameter <4 cm and discouraged the use of a No. 10 lymphadenectomy in the treatment of these tumors.^13^ Therefore, we evaluated the frequency of No. 10 LNM in Siewert type II and III AEG with tumor diameters of <4 cm and ≥4 cm in this study. Of the 36 patients with No. 10 LNM, only 1 had a tumor <4 cm in diameter, and the rest had tumors ≥4 cm in diameter. For Siewert type III AEG, the No. 10 LNM rate was significantly higher for tumors with a diameter ≥4 cm compared with those with a diameter <4 cm. This is because the anatomical position and biological behavior of Siewert type III AEG with a larger tumor diameter are quite similar to those of advanced proximal gastric cancer, which has a higher rate of No. 10 LNM.^24^ Tumor stage was more advanced and had a higher incidence of No. 10 LNM for a tumor diameter ≥4 cm because it is very difficult to perform a No. 10 lymphadenectomy but easy to damage the vessels and tissues in the splenic hilar area, which can induce more major complications for patients with a tumor diameter ≥4 cm.

Although there is a certain incidence of No. 10 LNM in Siewert type II and III AEG, the survival benefit of a No. 10 lymphadenectomy for Siewert type II and III AEG patients with a total gastrectomy is uncertain and controversial. A systematic review of spleen and pancreas preservation in an extended lymphadenectomy for advanced gastric cancer was presented by Brar et al.^25^ They concluded that the preservation of the spleen and pancreas during an extended lymphadenectomy would not decrease the OS. Goto et al^9^ evaluated 132 patients with Siewert type II/III AEG and found that a No. 10 lymphadenectomy did not increase the OS for Siewert type II/III AEG. Yang et al^26^ indicated that the 5-year survival rates of patients with Siewert type II and III AEG were 51.6% and 57.2% in the No. 10D+ group and 28.5% and 39.5% in the No. 10D– group, respectively. Although the difference in 5-year survival rates between the 2 groups did not reach statistical significance, the differences were obvious. Therefore, Yang et al recommended a No. 10 lymphadenectomy for Siewert type II/III AEG during a total gastrectomy. To estimate the therapeutic value of a No. 10 lymph node dissection, Hosokawa et al^27^ calculated the index of estimated benefit from lymph-node dissection^28^ and determined that a No. 10 lymphadenectomy would provide some survival benefit for Siewert type II/III AEG. The No. 10D+ group showed superior 3-year OS and DFS rates compared with the No. 10D– group. Moreover, in the stratified analysis according to the Siewert classification, the 3-year OS and DFS rates were similar between the No. 10D+ and No. 10D– groups for patients with Siewert type II AEG, but the No. 10D+ group showed significantly longer 3-year OS and DFS for patients with Siewert type III AEG. The new edition of the Japanese gastric cancer treatment guidelines^14^ showed that a No. 10 lymphadenectomy was unnecessary for patients with AEG with a tumor diameter <4 cm. However, the guideline did not offer relevant suggestions for AEG with a tumor diameter ≥4 cm. Whether a No. 10 lymphadenectomy should be performed for all Siewert type II/III AEG cases with a tumor diameter ≥4 cm is unknown. Fang et al^5^ also indicated that tumor diameter was an independent factor of survival after curative resection for Siewert type II/ III AEG. They found that patients with a tumor diameter ≥5 cm had a 1.99 times greater risk of death compared to those with a tumor diameter <5 cm after curative surgery. Our results showed that the No. 10D+ group had better 3-year OS and DFS rates for patients with Siewert type III AEG and a tumor diameter ≥4 cm. We believe that the obvious survival benefit of a No. 10 lymphadenectomy for Siewert type III AEG with a tumor diameter ≥4 cm is due to the much higher rate of No. 10 LNM. N stage was significantly more advanced when No. 10 LNM occurred. Siewert type III AEG with a tumor diameter ≥4 cm was associated with a higher incidence of No. 10 LNM and a more advanced N stage. Therefore, a No. 10 lymphadenectomy would significantly improve the prognosis for patients with Siewert type III AEG and a tumor diameter ≥4 cm. Furthermore, our study revealed that a No. 10 lymphadenectomy was an independent prognostic factor in Siewert type III AEG with a tumor diameter ≥4 cm.

To our knowledge, the present study is the first and largest to evaluate the oncologic outcomes of SPL for Siewert type II and III AEG based on tumor diameter. However, our study did have some limitations, including the use of data from a single center and its retrospective nature, which introduces a possible selection bias. The preoperative examination was unable to accurately assess the status of LNM in the splenic hilar. Therefore, a No. 10 lymphadenectomy should have been performed for some patients with No. 10 LNM, and the results of the metastatic ratio and survival differences may be biased. In addition, data to assess how survival could be attributed to a No. 10 LND versus chemotherapy were not available in our center. Therefore, in our future research, a further randomized prospective study is required to confirm our results, particularly for Siewert type II AEG, to provide evidence for the standardized treatment of AEG.

## References

[1] Vial M Grande L Pera M Epidemiology of adenocarcinoma of the esophagus, gastric cardia, and upper gastric third. *Recent Results Cancer Res* 2010; 182:1–17. 20676867 10.1007/978-3-540-70579-6_1

[2] Buas MF Vaughan TL Epidemiology and risk factors for gastroesophageal junction tumors: understanding the rising incidence of this disease. *Semin Radiat Oncol* 2013; 23:3–9. 23207041 10.1016/j.semradonc.2012.09.008 PMC3535292

[3] Siewert JR Stein HJ Classification of adenocarcinoma of the oesophagogastric junction. *Br J Surg* 1998; 85:1457–1459. 9823902 10.1046/j.1365-2168.1998.00940.x

[4] Meier I Merkel S Papadopoulos T Adenocarcinoma of the esophagogastric junction: the pattern of metastatic lymph node dissemination as a rationale for elective lymphatic target volume definition. *Int J Radiat Oncol Biol Phys* 2008; 70:1408–1417. 18374226 10.1016/j.ijrobp.2007.08.053

[5] Fang WL Wu CW Chen JH Esophagogastric junction adenocarcinoma according to Siewert classification in Taiwan. *Ann Surg Oncol* 2009; 16:3237–3244. 19636628 10.1245/s10434-009-0636-9

[6] Hasegawa S Yoshikawa T Rino Y Priority of lymph node dissection for Siewert type II/III adenocarcinoma of the esophagogastric junction. *Ann Surg Oncol* 2013; 20:4252–4259. 23943020 10.1245/s10434-013-3036-0

[7] Goto H Tokunaga M Sugisawa N Value of splenectomy in patients with Siewert type II adenocarcinoma of the esophagogastric junction. *Gastric Cancer* 2013; 16:590–595. 23179369 10.1007/s10120-012-0214-x

[8] Chen XZ Zhang WH Hu JK Lymph node metastasis and lymphadenectomy of resectable adenocarcinoma of the esophagogastric junction. *Chin J Cancer Res* 2014; 26:237–242. 25035648 10.3978/j.issn.1000-9604.2014.06.17 PMC4076724

[9] Goto H Tokunaga M Miki Y The optimal extent of lymph node dissection for adenocarcinoma of the esophagogastric junction differs between Siewert type II and Siewert type III patients. *Gastric Cancer* 2014. 10.1007/s10120-014-0364-0 PMC4371819 24658651

[10] Hartgrink HH Should we remove splenic hilus lymph nodes for esophagogastric junction adenocarcinoma? *Gastric Cancer* 2013; 16:454–456. 23417699 10.1007/s10120-013-0237-y

[11] Kunisaki C Makino H Takagawa R Tumor diameter as a prognostic factor in patients with gastric cancer. *Ann Surg Oncol* 2008; 15:1959–1967. 18369676 10.1245/s10434-008-9884-3

[12] Li C Oh SJ Kim S Risk factors of survival and surgical treatment for advanced gastric cancer with large tumor size. *J Gastrointest Surg* 2009; 13:881–885. 19184612 10.1007/s11605-009-0800-3

[13] Japanese Gastric Cancer Association. Japanese gastric cancer treatment guidelines (ver. 4) [M]. *Tokyo: Kanehara* 2014; 34–41.

[14] Dindo D Demartines N Clavien PA Classification of surgical complications: a new proposal with evaluation in a cohort of 6336 patients and results of a survey. *Ann Surg* 2004; 240:205–213. 15273542 10.1097/01.sla.0000133083.54934.ae PMC1360123

[15] Edge SB Compton CC The American Joint Committee on Cancer: the 7th edition of the AJCC cancer staging manual and the future of TNM. *Ann Surg Oncol* 2010; 17:1471–1474. 20180029 10.1245/s10434-010-0985-4

[16] Jia-Bin W Chang-Ming H Chao-Hui Z Laparoscopic spleen-preserving No. 10 lymph node dissection for advanced proximal gastric cancer in left approach: a new operation procedure. *World J Surg Oncol* 2012; 10:241. 23146045 10.1186/1477-7819-10-241 PMC3502297

[17] Zheng CH Huang CM Li P Laparoscopic spleen-preserving hilar lymph nodes dissection based on splenic hilar vascular anatomy. *Zhonghua Xiaohua Waike Zazhi* 2012; 11:215–219.

[18] Huang CM Chen QY Lin JX Huang's three-step maneuver for laparoscopic spleen-preserving No. 10 lymph node dissection for advanced proximal gastric cancer. *Chin J Cancer Res* 2014; 26:208–210. 24826062 10.3978/j.issn.1000-9604.2014.04.05 PMC4000903

[19] Japanese classification of gastric carcinoma: 3rd English edition. *Gastric Cancer* 2011; 14:101–112. 21573743 10.1007/s10120-011-0041-5

[20] Kurokawa Y Sasako M Sano T Ten-year follow-up results of a randomized clinical trial comparing left thoracoabdominal and abdominal transhiatal approaches to total gastrectomy for adenocarcinoma of the oesophagogastric junction or gastric cardia. *Br J Surg* 2015; 102:341–348. 25605628 10.1002/bjs.9764 PMC5024022

[21] Kakeji Y Yamamoto M Ito S Lymph node metastasis from cancer of the esophagogastric junction, and determination of the appropriate nodal dissection. *Surg Today* 2012; 42:351–358. 22245924 10.1007/s00595-011-0114-4

[22] Kunisaki C Makino H Suwa H Impact of splenectomy in patients with gastric adenocarcinoma of the cardia. *J Gastrointest Surg* 2007; 11:1039–1044. 17514409 10.1007/s11605-007-0186-z

[23] Shin SH Jung H Choi SH Clinical significance of splenic hilar lymph node metastasis in proximal gastric cancer. *Ann Surg Oncol* 2009; 16:1304–1309. 19241107 10.1245/s10434-009-0389-5

[24] Monig SP Collet PH Baldus SE Splenectomy in proximal gastric cancer: frequency of lymph node metastasis to the splenic hilus. *J Surg Oncol* 2001; 76:89–92. 11223832 10.1002/1096-9098(200102)76:2<89::aid-jso1016>3.0.co;2-i

[25] Brar SS Seevaratnam R Cardoso R A systematic review of spleen and pancreas preservation in extended lymphadenectomy for gastric cancer. *Gastric Cancer* 2012; 15 suppl 1:S89–99. 21915699 10.1007/s10120-011-0087-4

[26] Yang K Zhang WH Chen XZ Survival benefit and safety of no. 10 lymphadenectomy for gastric cancer patients with total gastrectomy. *Medicine* 2014; 93:e158. 25437029 10.1097/MD.0000000000000158 PMC4616371

[27] Hosokawa Y Kinoshita T Konishi M Clinicopathological features and prognostic factors of adenocarcinoma of the esophagogastric junction according to Siewert classification: experiences at a single institution in Japan. *Ann Surg Oncol* 2012; 19:677–683. 21822549 10.1245/s10434-011-1983-x

[28] Sasako M McCulloch P Kinoshita T New method to evaluate the therapeutic value of lymph node dissection for gastric cancer. *Br J Surg* 1995; 82:346–351. 7796005 10.1002/bjs.1800820321

